# Effects of Intermittent Normobaric Hypoxia on Health-Related Outcomes in Healthy Older Adults: A Systematic Review

**DOI:** 10.1186/s40798-023-00560-0

**Published:** 2023-02-26

**Authors:** Rafael Timon, Ismael Martinez-Guardado, Franck Brocherie

**Affiliations:** 1 Sport Sciences Faculty, Universidad de Extremadura, Av/ Universidad s/n, 10004 Cáceres, Spain; 2 BRABE Group. Faculty of Life and Nature Sciences, Universidad de Nebrija, Madrid, Spain; 3 Laboratory Sport, Expertise and Performance (EA 7370), French Institute of Sport (INSEP), Paris, France

**Keywords:** Normobaric hypoxia, Elderly, Bone health, Cardiovascular health, Metabolic health, Functional fitness

## Abstract

**Background:**

Aging is a degenerative process that is associated with an increased risk of diseases. Intermittent hypoxia has been investigated in reference to performance and health-related functions enhancement. This systematic review aimed to summarize the effect of either passive or active intermittent normobaric hypoxic interventions compared with normoxia on health-related outcomes in healthy older adults.

**Methods:**

Relevant studies were searched from PubMed and Web of Science databases in accordance with PRISMA guidelines (since their inceptions up until August 9, 2022) using the following inclusion criteria: (1) randomized controlled trials, clinical trials and pilot studies; (2) Studies involving humans aged > 50 years old and without any chronic diseases diagnosed; (3) interventions based on in vivo intermittent systemic normobaric hypoxia exposure; (4) articles focusing on the analysis of health-related outcomes (body composition, metabolic, bone, cardiovascular, functional fitness or quality of life). Cochrane Collaboration recommendations were used to assess the risk of bias.

**Results:**

From 509 articles initially found, 17 studies were included. All interventions were performed in moderate normobaric hypoxia, with three studies using passive exposure, and the others combining intermittent hypoxia with training protocols (*i.e.,* using resistance-, whole body vibration- or aerobic-based exercise).

**Conclusions:**

Computed results indicate a limited effect of passive/active intermittent hypoxia (ranging 4–24 weeks, 2–4 days/week, 16–120 min/session, 13–16% of fraction of inspired oxygen or 75–85% of peripheral oxygen saturation) compared to similar intervention in normoxia on body composition, functional fitness, cardiovascular and bone health in healthy older (50–75 years old) adults. Only in specific settings (*i.e.,* intermediate- or long-term interventions with high intensity/volume training sessions repeated at least 3 days per week), may intermittent hypoxia elicit beneficial effects. Further research is needed to determine the dose–response of passive/active intermittent hypoxia in the elderly.

*Trial registration*. Systematic review registration: PROSPERO 2022 CRD42022338648.

## Key Points

- Passive and active intermittent normobaric hypoxia has a limited effect on health-related outcomes in healthy older adults, compared to similar intervention in normoxia.
- Intermediate- or long-term intermittent hypoxia interventions with high intensity/volume training sessions repeated at least 3 days per week could have a putative effect on functional fitness and fat mass loss compared to normoxia.
- More research is needed to determine the dose–response of passive/active intermittent normobaric hypoxia in healthy older adults

## Introduction

Aging is a degenerative process produced as a result of different cellular dysfunctions and tissue damages, which cause a gradual loss of physical and mental capacities [1]. This progressive deterioration has been associated with the development of age-related disorders [2]. To circumvent them, different strategies, such as individualized socio-health care, the use of medications, exercise, maintaining healthy lifestyles or improving the psycho-social environment of people, have been proposed [3, 4].

Currently, some review studies have proposed exposure to hypoxic conditions, either passive or active in combination with exercise, as a promising tool to achieve health benefits [5–8], targeting hypoxia-inducible factor (HIF) and its signaling pathway as a novel therapeutic option to deal with various chronic diseases [9, 10]. On the one hand, an oxygen-deprived (hypoxia) environment impairs cell adaptation and survival [11] with chronic exposure to severe hypoxia leading to hypoxemia and cardiovascular and pulmonary complications [12, 13]. On the other hand, intermittent hypoxia (IH) has been shown to exert beneficial effects at the cardiovascular, metabolic, and cognitive levels, both in healthy and pathological individuals [14–16].

IH could be defined as repeated exposure to hypoxia interspersed with normoxia. In the clinical setting, IH has been associated with obstructive sleep apnea syndrome (OSAS), a disorder of sleep breathing characterized by nightly high frequency repetitive and prolonged periods of complete or partial obstruction of the upper airway [17, 18] that causes multiple alterations and pathologies in individuals [19, 20]. However, such chronic, severe, and repetitive IH must be differentiated from IH with controlled reduction of oxygen. OSAS causes a dysregulated transcription of HIF-1α and HIF-2 α, increasing HIF-1α and decreasing HIF-2α [21]. On the contrary, IH and controlled reoxygenation prevents the hydroxylation and degradation of HIF-1α, allowing its stabilization and entry into the cell nucleus to activate genetic transcription factors related to erythropoiesis, osteogenesis, angiogenesis, lipolysis, and antioxidant capacity [22–24]. For instance, short-term daily IH sessions consisting of 3–4 bouts of 5–7 min exposure to 10–12% of fraction of inspired oxygen (%FiO_2_) alternated with similar periods of normoxia (%FiO_2_ 21%) for at least 2–3 weeks have been shown to be beneficial for cardiovascular, respiratory and neurological disorders [25, 26].

Although previous studies have suggested that IH could have positive effects on hypertension, hemodynamics, neurodegeneration, and obesity [6, 7, 14], the current scientific knowledge is not unanimous. The physiological and metabolic adaptations induced by IH could depend on the hypoxic dose (*i.e.*, severity, duration, and exposure time of the intervention), as well as other factors such as genetics, age of the individuals or training status [27–29]. To date, there is no systematic review in the present scientific literature that specifically examines whether IH has positive or negative effects on health-related outcomes in healthy older adults. We therefore summarize the effect of either passive or active IH interventions compared with normoxia on health-related outcomes in healthy older adults.

## Methods

### Search Strategy

A systematic review was performed following the Preferred Reporting Guidelines for Systematic Reviews and Meta-analyzes (PRISMA) [30]. The systematic review was registered using the PROSPERO International database of systematic review protocols (Registration number: PROSPERO 2022 CRD42022338648). Randomized controlled trials (RCTs), clinical trials and pilot studies were identified by electronically searching the following databases: PubMed (MEDLINE) and Web of Science (WoS), and through manual searching of reference lists of eligible studies. To optimize the identification of relevant articles, the terms “intermittent hypoxia”, “hypoxic conditioning”, “normobaric hypoxia”, “elderly”, “older adults”, “therapeutic” and “health benefits” were combined with Boolean operators (“AND” and “OR”) and searched from inception up until August 9, 2022. All references were extracted and imported into an open-source research tool to systematize studies.

### Inclusion Criteria

1. *Types of studies*. Articles published in peer-reviewed journals written in English. Randomized controlled trials (RCTs), clinical trials and pilot studies, comparing normobaric hypoxia *vs*. normoxia, were included.
2. *Type of participants*. Articles with humans (females and males) over 50 years old who had not been diagnosed with chronic diseases.
3. *Types of interventions*. To be included in this systematic review, interventions had to use only in vivo systemic IH training. Protocols using hypobaric hypoxia, hyperoxia or blood flow restriction (or other combination) were excluded, as well as acute or single IH trials.
4. *Types of outcomes evaluated*. Articles that focused on the analysis of health-related variables (*i.e.*, metabolic, body composition, bone, cardiovascular, functional fitness or quality of life) were included.

### Selection Process

Two investigators (RT and FB) selected the eligible articles based on title, abstract and full paper, using the inclusion criteria. Disagreements were resolved by consensus. Steps followed in the selection of studies were: (1) identification of potential studies, (2) duplicates removal, (3) title and abstract examination, (4) full text exploration and (5) checking of the quality of research and relevance to the purpose of the review.

### Risk of Bias Assessment

Cochrane Collaboration recommendations [31] for systematic reviews of interventions were used to assess the risk of bias for all articles. Investigators' assessments were classified as *'low risk'*, *'high risk'* or *'unclear risk'* of bias, referring to the following domains: random sequence generation, allocation concealment, blinding of participants and personnel, blinding of outcome assessments, incomplete outcome data and selective reporting. Two investigators (RT and FB) independently assessed the methodological quality of all articles selected. The discrepancies were resolved with the conciliation work carried out by another investigator (IMG).

### Data Extraction

Data extraction was completed by the lead investigator (RT) who compiled them into descriptive tables. The data extraction was subsequently checked by another investigator (IMG). The following data were extracted for each category: (a) author, year of publication, (b) participants, (c) experimental groups, (d) intervention, (e) duration/frequency (f) %FiO_2_ or % peripheral oxygen saturation (%SpO_2_), (g) health-related outcomes.

## Results

### Search Results

**Fig. 1:**
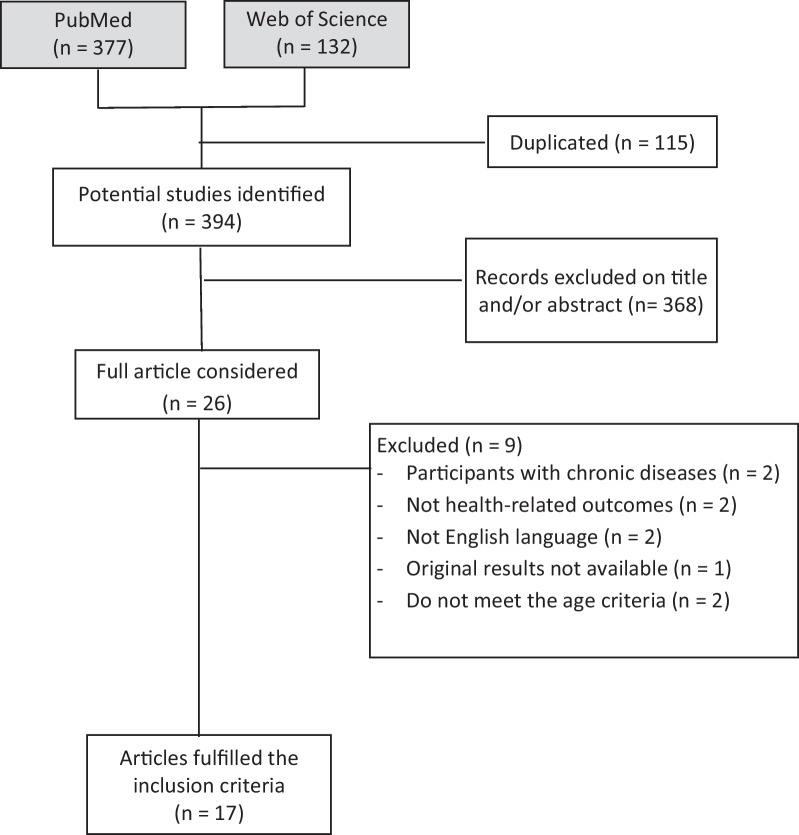
Flowchart of the study selection

The process of identifying eligible studies is shown in Fig. 1. Five hundred and nine records were initially identified through the databases. One hundred and fifteen articles were removed for being duplicates. Of the rest of the identified manuscripts, 26 potentially eligible articles were included based on their title and abstract. After full text exploration, only 17 articles fulfilled the inclusion criteria. The characteristics of each included study are described in Table 1.

**Table 1:** Characteristics of included studies

Study	Participants	Experimental groups	Intervention	Duration/ Frequency	FiO_2_ or SpO_2_	Health-related outcomes
Allsopp et al. [40]	n = 20 (12 males; 8 females) age: 60–75 yr	Normoxia + exercise (n = 10) Hypoxia + exercise (n = 10)	Resistance training (4 exercises at 70% 1RM with 4 × 10 reps)	8 weeks; 2 days per week; ~ 60 min per session	Normobaric hypoxia 14.4%	→Glucose →Insulin →HOMA-IR
Allsopp et al. [41]	n = 20 (12 males; 8 females) age: 60–75 yr	Normoxia + exercise (n = 10) Hypoxia + exercise (n = 10)	Resistance training (4 exercises at 70% 1RM with 4 × 10 reps)	8 weeks; 2 days per week; ~ 60 min per session	Normobaric hypoxia 14.4%	→Lean mass →Fat mass →Glucose → CHO →Systolic BP
Camacho-Cardenosa et al. [44]	n = 19 (8 males; 11 females) age: 65–75 yr	Normoxia + exercise (n = 10) Hypoxia + exercise (n = 9)	WBV training (12.6 Hz, 4 mm) (4 sets × 30 s, 60-s rest)	18 weeks; 2 days per week; 16 min per session	Normobaric hypoxia 16.1%	→Leg lean mass →Functional mobility (TUGT)
Camacho-Cardenosa et al. [35]	n = 10 age: ≥ 75 yr	Control, no intervention (n = 5) Hypoxia (n = 5)	Passive hypoxic exposure	18 weeks; 2 days per week; 16 min per session	Normobaric hypoxia 16.1%	→Body fat mass →Trunk fat mass →Femoral BMD →Trochanter BMD ↑Whole body BMD
Camacho-Cardenosa et al. [43]	n = 31 (11 males, 20 females) age: ≥ 65–75 yr	Control, no intervention (n = 10) Normoxia + exercise (n = 11) Hypoxia + exercise (n = 10)	WBV training (12.6 Hz, 4 mm) (4 sets × 30 s, 60-s rest)	18 weeks; 2 days per week; 16 min per session	Normobaric hypoxia 16.1%	→Body lean mass →Maximal strength →Endurance strength
Camacho-Cardenosa et al. [40]	n = 30 (10 males, 20 females) age: ≥ 65–75 yr	Control, no intervention (n = 10) Normoxia + exercise (n = 10) Hypoxia + exercise (n = 10)	WBV training (12.6 Hz, 4 mm) (4 sets × 30 s, 60-s rest)	18 weeks; 2 days per week; 16 min per session	Normobaric hypoxia 16.1%	→Whole body BMD →Femoral BMD →Trochanter BMD
Chacaroun et al.[37]	n = 23 (19 males; 4 females) age: 54 ± 11 yr	Normoxia + exercise (n = 11) Hypoxia + exercise (n = 12)	Submaximal constant-load exercise in cycle ergometer (75% HR_max_)	8 weeks; 3 days per week; 45 min per session	Normobaric hypoxia 13%	→Glucose →Insulin →HOMA2-IR →CHO →Triglycerides →HDL →Lean mass →Fat mass →Systolic BP
Chacaroun et al. [36]	n = 35 (24 males, 11 females) age: 54 ± 9.3 yr	Normoxia, sham condition (n = 11) Sustained hypoxia (n = 13) Intermittent hypoxia (n = 12)	Passive hypoxic exposure	8 weeks; 3 days per week; Sustained hypoxia: 60 min per session Intermittent hypoxia: 7 cycles (5 min hypoxia + 3 min normoxia) per session	Normobaric hypoxia FiO2 based on SpO_2_ = 75%	→Systolic BP ↓Diastolic BP →Vascular function (PWV) →HOMA-IR →Glucose →Lipid profile →CRP
Chobanyan-Jürgens et al. [46]	n = 29 (15 males, 14 females) age: 55–75 yr	Normoxia + exercise (n = 15) Hypoxia + exercise (n = 14)	Submaximal bicycle exercise at 60–70% VO_2_max	8 weeks; 3 days per week; 30–40 min per session	Normobaric hypoxia 15%	→Insulin sensivity index →HOMA-IR →Waist circumference →%Fat mass →CHO →GLUT4
Hein et al. [45]	n = 29 (15 males, 14 females) age: 55–75 yr	Normoxia + exercise (n = 15) Hypoxia + exercise (n = 14)	Submaximal bicycle exercise at 60–70% VO_2_max	8 weeks; 3 days per week; 30–40 min per session	Normobaric hypoxia 15%	→Body fat mass →VO_2_max →Rest BP →Exercise BP
Park et al. [47]	n = 24 (men) age: 65–70 yr	Normoxia + exercise (n = 12) Hypoxia + exercise (n = 12)	Aerobic exercise (60–70% HR_max_) on treadmill (30 min) and bicycle (30 min) + elastic resistance training (6 exercises, 3 × 10–15 reps; RPE: 6–7 out of 10; 30–45 min)	12 weeks; 3 days per week; 90–120 min per session	Normobaric hypoxia 14.5%	↑Fat-free mass ↓Body fat mass ↑Physical fitness ↑FVC ↑FEV_1_ ↑MVV ↑HRV (high frequency)
Schega et al.[39]	n = 36 (19 males, 17 females) age: 60–75 yr	Normoxia + exercise (n = 18) Hypoxia + exercise (n = 18)	Passive hypoxia exposure (90 min) + aerobic exercise on bicycle (30 min, 70–75% HR_max_)	4 weeks; 3 days per week; 120 min per session	Normobaric hypoxia FiO_2_ based on SpO_2_ = 80%	→BDNF →VO_2_max →Cognitive function (Stroop test) ↑Hb; Hct ↑Red-blood cells
Schega et al.[38]	n = 34 (8 males; 16 females) age: 60–70 yr	Normoxia + exercise (n = 17) Hypoxia + exercise (n = 17)	Passive hypoxic exposure (60 min) + full-body strength-endurance (30 min, 5 exercises at 50% 1RM with 2 × 25 reps)	6 weeks; 3 days per week; 90 min per session	Normobaric hypoxia FiO_2_ based on SpO_2_ = 80%	↑Attention (d2 test) ↑Sleep quality (PSQI test) →Speed of cognitive performance (ZVT test) →Quality of life (physical and mental component) →Strength-endurance capacity
Timon et al. [32]	n = 38 (15 males; 23 females) age: 65–75 yr	Control, no intervention (n = 19) Hypoxia (n = 19)	Passive hypoxic exposure	24 weeks; 3 days per week; 45 min per session	Normobaric hypoxia 16.1%	→Lean mass ↓Body fat mass →Whole body BMD →Whole body BMC →Systolic BP →Glucose → CHO →Triglycerides CRP ↑PINP b-CTX →Interleukin 8 →Interleukin 10
Timon et al. [33]	n = 54 (22 males; 32 females) age: 65–75 yr	Control, no intervention (n = 19) Normoxia + exercise (n = 18) Hypoxia + exercise (n = 17)	Resistance training (9 exercises with elastic bands and kettlebells; 3 sets × 12–15 reps)	24 weeks; 3 days per week; 45 min per session	Normobaric hypoxia 16.1%	→Lean mass →Fat mass →Functional fitness (SFT) →Balance →Fear of falling
Timon et al. [34]	n = 54 (22 males; 32 females) age: 65–75 yr	Control, no intervention (n = 19) Normoxia + exercise (n = 18) Hypoxia + exercise (n = 17)	Resistance training (9 exercises with elastic bands and kettlebells; 3 sets × 12–15 reps)	24 weeks; 3 days per week; 45 min per session	Normobaric hypoxia 16.1%	→CRP →Interleukin 6 →Interleukin 8 →Interleukin 10 →VCAM1
Torpel et al.[29]	n = 36 (18 males; 18 females) age: 60–75 yr	Normoxia + exercise (n = 17) Hypoxia + exercise (n = 19)	Resistance training (circuit with 8 machine-based resistance exercises at 25–40% 1RM; 3 × 15 reps); RPE: 7 out of 10	5 weeks; 4 days per week; ~ 60 min per session	Normobaric hypoxia FiO_2_ based on SpO_2_ ranging 80–85%	→Fat mass →Fat-free mass →VO_2_max → Muscular strength →BP →Hb →Erythropoietin →Blood volume

↑ Significant increase in the study variable; ↓ Significant decrease in the study variable; → The study variable remains unchanged

1RM: one-repetition maximum; b-CTX: Beta C-terminal telopeptide of collagen; BDNF: brain-derived neurotrophic factor; BMC: bone mineral content; BMD: bone mineral density; BP: blood pressure; CHO: cholesterol; CRP: C-reactive protein; FEV_1_: forced expiratory volume in 1 s; FiO2: fraction of inspired oxygen, FVC: forced vital capacity; GLUT4: glucose transporter 4; Hb: hemoglobin; Hct: hematocrit; HDL: high-density lipoprotein; HOMA-IR: homeostatic model assessment-insulin resistance; HRmax: maximum heart rate; HRV: Heart rate variability; MVV: maximum voluntary ventilation; PINP: N-terminal propeptide of type I procollagen; PWV: pulse wave-velocity; RPE: rating of perceived exertion; SFT: senior fitness test; SpO_2_: peripheral oxygen saturation; TUGT: timed up and go test; VCAM1: vascular cell adhesion molecule 1; VO_2_max: maximal oxygen uptake; WBV: whole body vibration

### Risk of Bias of Included Studies

According to the Cochrane Collaboration recommendations, the results of the risk of bias assessment showed that 9 out of 17 articles had a *low risk* of bias in all domains. All articles had a *low risk* of allocation concealment and blinding of outcomes bias. Each article was described as randomized, but the randomization method was unclear for one study [32]. Two studies revealed *high risk* of blinding participants and personnel bias [33, 34], and in two other studies, the information about the blinding was *unclear* [32, 35]. Incomplete outcome data were at a *low risk* of bias in all articles, except for two studies [35, 36] that were at *high* risk (exclusions were not defined from the analysis and lack of several relevant statistics). Moreover, the results for selective reporting bias were at *high* risk for four studies [37–39] (conclusions were only partially supported by the findings presented). The risk of bias assessment of all included studies is displayed in Table 2.

**Table 2:** Risk of bias assessment of included studies

Study	Random sequence	Allocation concealment	Blinding of participants/personnel	Blinding of outcomes	Incomplete outcome data	Selective reporting
Allsopp et al. [40]	Low	Low	Low	Low	Low	Low
Allsopp et al. [41]	Low	Low	Low	Low	Low	Low
Camacho-Cardenosa et al. [44]	Low	Low	Low	Low	Low	Low
Camacho-Cardenosa et al. [35]	Low	Low	Unclear	Low	High	Low
Camacho-Cardenosa et al. [43]	Low	Low	Low	Low	Low	Low
Camacho-Cardenosa et al. [42]	Low	Low	Low	Low	Low	Low
Chacaroun et al. [37]	Low	Low	Low	Low	Low	High
Chacaroun et al. [36]	Low	Low	Low	Low	High	High
Chobanyan-Jürgens et al. [46]	Low	Low	Low	Low	Low	Low
Hein et al. [45]	Low	Low	Low	Low	Low	Low
Park et al. [47]	Low	Low	Low	Low	Low	Low
Schega et al. [39]	Low	Low	Low	Low	Low	High
Schega et al. [38]	Low	Low	Low	Low	Low	High
Timon et al. [32]	Unclear	Low	Unclear	Low	Low	Low
Timon et al. [33]	Low	Low	High	Low	Low	Low
Timon et al. [34]	Low	Low	High	Low	Low	Low
Torpel et al. [29]	Low	Low	Low	Low	Low	Low

### Characteristics of Studies and Interventions

Fourteen studies [29, 33, 34, 36, 37, 39–47] were classified as RCTs, one study [32] as a quasi-experimental clinical trial, and two studies [35, 38] were described as pilot studies. Only one study [47] had a sample including solely males (n = 24), while the remaining investigations used mixed samples (n ranging 8–22 males and 4–32 females, respectively). The duration of the interventions varied widely, from short-term (4–6 weeks) [29, 38, 39], to intermediate (8–12 weeks) [36, 37, 40, 41, 45–47] and long-term duration (18–24 weeks) [32–35, 42–44], with a frequency of 2–4 sessions per week. The duration of the sessions was also very wide-ranging, from 16-min per session [35, 42–44] up to 90–120 min per session [38, 39, 47], and with intermediate durations of 30–60 min per session [29, 32–34, 36, 37, 40, 41, 45, 46]. All interventions were performed under moderate normobaric hypoxia, with %FiO_2_ ranging 13.0–16.1% or %SpO_2_ ranging 75–85%. Among all these interventions, three studies proposed the exclusive use of passive IH [32, 35, 36], but the rest of the studies (n = 14) combined IH with exercise training, either sequentially [38, 39] or simultaneously [29, 33, 34, 37, 40–47]. Six studies carried out resistance training programs with full-body routines (4 to 8 exercises) within the session, which were performed with strength training machines [29, 38, 40, 41] or elastic bands and kettlebells [33, 34]. Four studies used a stable-load, submaximal-intensity aerobic cycling training program [37, 39, 45, 46]. In one study [47], participants performed aerobic exercises and resistance exercises with elastic bands within the same session. Finally, in three studies [42–44], all conducted by the same author group, whole-body vibration (WBV) training was performed on a vibrating platform at an intensity of 12.6 Hz and an amplitude of 4 mm.

### Effects of IH Interventions Compared with Normoxia on Health-Related Outcomes

In the included studies, the different health-related outcomes investigated included metabolic [32, 36, 37, 40, 41, 46], body composition [29, 32, 33, 35, 37, 41, 43–47], blood pressure [32, 36, 37, 41, 45], bone [32, 35, 42], inflammatory [32, 34, 36] and hematological [29, 39] parameters, as well as functional fitness [29, 33, 38, 39, 43–45, 47] or parameters related to quality of life [38, 39]. The main findings of the studies considered for review are summarized in Table 1.

### Metabolic Parameters

Six of the seventeen studies evaluated metabolic parameters [32, 36, 37, 40, 41, 46] such as glucose, total and HDL-cholesterol, triglycerides, glucose transporter 4 (GLUT4), insulin, insulin sensitivity index and the homeostatic model assessment-insulin resistance (HOMA-IR). None of the studies showed significantly improved values after an IH intervention compared to normoxia, either using a passive [32, 36] or active [37, 40, 41, 46] paradigm.

### Body Composition

Eleven studies included body composition parameters in their assessments, albeit using different technologies: one study [37] used whole body magnetic resonance imagery (MRI) to determine fat and lean mass, two studies [29, 47] applied bioelectrical impedance to assess fat mass and fat-free mass, two other studies [45, 46] determined body fat and fat-free mass by air-displacement plethysmography, and six studies [32, 33, 35, 41, 43, 44] used dual-energy X-ray absorptiometry (DXA) to determine lean mass, fat mass or bone mineral content (BMC), from the whole body or specific areas. None of the active IH interventions using resistance training, aerobic training or WBV had any added benefit on lean mass, fat mass or BMC compared to similar intervention in normoxia, with the exception of the study by Park et al. [47] who observed increases in fat-free mass and decreases in body fat percentage. In this study, unlike all the others, long multimodal training sessions (90–120 min) were used, with a FiO_2_ of 14.5%, combining aerobic exercise on treadmill (30 min) then on bicycle (30 min) followed by elastic resistance exercises (30–45 min). Regarding the two studies that used passive IH, only one [32] obtained decreases of body fat mass, without changes in body lean mass, after a 24-week intervention with three sessions per week of 45 min at FiO_2_ of 16.1%.

### Blood Pressure

The effect of IH on blood pressure was investigated in six studies [29, 32, 36, 37, 41, 45]. In general, either passive [*i.e.*, over 24 weeks [32]] or active IH [i.e., 5–8 weeks of resistance training [29, 41] or aerobic training [37, 45]], did not produce greater benefit than normoxic intervention on blood pressure values in healthy older adults. Only one study [36] using a 8-week passive hypoxia (SpO_2_ = 75%) intervention showed a significant decrease in diastolic blood pressure compared to the normoxia intervention.

### Bone Parameters

Bone mineral density (BMD) (from whole body or specific areas) was assessed in three studies [32, 35, 42], with additional dynamic biomarkers related to bone remodeling [N-terminal propeptide of type I procollagen (PINP) and beta C-terminal telopeptide of collagen b-CTX)] evaluated in one of them [32]. Two of these studies were carried out with passive IH interventions [32, 35], while the third one used active IH WBV training [42]. Eighteen weeks of IH WBV training (%FiO_2_ 16.1%) did not lead to significant changes compared to the normoxic group in whole body BMD, femoral BMD and trochanter BMD [42]. However, passive IH sessions of 16–45 min over 18–24 weeks at %FiO_2_ of 16.1% induced changes in whole body BMD [35] and in PINP (bone formation biomarker) and b-CTX (bone resorption biomarker) in sedentary older adults [32] compared to similar intervention in normoxia.

### Inflammatory Biomarkers

Only three studies, using passive [32, 36] and active IH [34] investigated the influence of such hypoxic intervention on inflammatory biomarkers. Reportedly, a 24-weeks IH (%FiO_2_ 16.1%) intervention including resistance training with elastic bands and kettlebells did not affect C-reactive protein, vascular cell adhesion molecule 1, and interleukins 6, 8 and 10 in comparison with similar intervention in normoxia [34]. Similarly, an 8-week intervention of passive hypoxia (SpO_2_ = 75%) also did not cause significant changes in C-reactive protein levels [36]. On the contrary, passive IH exposure at FiO2 of 16.1% (*i.e.*, 24 weeks) induced a decrease in C-reactive protein compared to a normoxic control *(i.e.,* no intervention) group [32].

### Hematological Parameters

In two studies [29, 39], variations in hematological parameters were evaluated after 4–5 weeks of active IH in healthy older adults. Normobaric hypoxic dose was tailored based on an SpO_2_ target of 80–85% in both experiments. Increases in red-blood cells, hemoglobin and hematocrit were observed after twelve sessions (120 min) that combined IH (90 min) with aerobic cycling exercise (30 min) [39]. However, when using resistance training (*i.e.,* 20 sessions of 60 min) [29], no changes in hemoglobin, blood volume and erythropoietin were observed.

### Functional Fitness and Pulmonary Capacity

A total of eight studies [29, 33, 38, 39, 43–45, 47] evaluated the functional fitness of older adults. Different physical capacities were measured using various tests. One study [47] evaluated pulmonary function [*i.e.*, forced vital capacity (FVC), forced expiratory volume in 1 s (FEV_1_), and maximal voluntary ventilation (MVV)] with a spirometer, two studies [29, 43] assessed muscle force parameters using an isokinetic dynamometer, three studies [29, 39, 45] measured maximal oxygen uptake (VO_2_max) by respiratory gas exchange systems, and four studies [33, 38, 44, 47] used functional tests (*i.e.*, senior fitness test, timed up and go test, one leg standing, pegboard) and exercise strength (*i.e.*, grip strength, crutches, push-ups). A 12-week IH intervention using moderate hypoxia (FiO_2_ 14.5%) with 90–120 min of multimodal training sessions (aerobic exercise and elastic resistance training) showed greater improvement in pulmonary capacity (FVC, FEV_1_ and MVV) and functional fitness (*i.e.*, chair stand, pegboard, tandem test and one leg standing) than normoxic training in men [47]. With the exception of one study [47], no studies found that active IH interventions, either with resistance [29, 33, 38], aerobic [39, 45] or WBV [43, 44] training, had an added positive effect on the physical fitness of healthy older adults compared to similar interventions performed in normoxia.

### Quality of Life

Two studies [38, 39] evaluated the effect of IH on characteristics related to quality of life and cognitive function. In both cases, the level of hypoxia was based on an SpO_2_ target of 80%. After a 6-week intervention with 90-min sessions combining passive IH (60 min) and active IH (30 min of strength-endurance exercises), improvements were only observed in attention levels and quality of sleep compared to the normoxic intervention, but not in speed of cognitive performance or on a quality of life questionnaire [38]. Similarly, after 4 weeks (3 sessions per week) of a combined intervention consisting of 90 min of passive IH followed by 30 min of active IH in each session, no changes were observed in cognitive function or in brain-derived neurotrophic factor (BDNF), a key molecule for long-term memory and neurogenesis stimulation [39].

## Discussion

This systematic review identified seventeen studies investigating the effects of IH on health-related parameters in healthy older adults in comparison with similar intervention in normoxia. Three studies [32, 35, 36] proposed passive IH interventions, and all others used active IH [29, 33, 34, 37–47]. The interventions were performed in normobaric hypoxia—%FiO_2_ ranging 13.0–16.1% or %SpO_2_ ranging 75–85%—corresponding to moderate altitude level; reason why none of the studies reported any complication or serious adverse effects in the participants despite their age. The most frequently investigated health-related outcomes were body composition [29, 32, 33, 35, 37, 41, 43–47], functional fitness [29, 33, 38, 39, 43–45, 47] and blood pressure [29, 32, 36, 37, 41, 45].

Overall, the results of this systematic review indicate that passive or active normobaric IH would have a limited positive effect on health-related outcomes in healthy older adults compared to similar intervention in normoxia, without any observed adverse effects in the participants. These findings differ from two recent reviews [48, 49] indicating that intermittent hypoxia-hyperoxia protocols could be effective at the cardiovascular, metabolic, and cognitive levels in an elderly population suffering from various diseases. The free radical signaling during hypoxia-hyperoxia interventions may lead to better induction of antioxidant synthesis than IH only [25]. Moreover, patients with coronary artery diseases showed a greater trend of change in hematological parameters and an enhanced erythropoietic response than healthy older adults [50], which may result in a different hypoxemic response and oxyhemoglobin dissociation curve between healthy and pathological older adults. Likewise, it was observed that HIF-1α mRNA expression after a 3-week intervention of passive IH (%FiO_2_ 12%) was increased in pre-diabetic individuals, but not in healthy older adults (40–70 years old) [51]. Without considering possible effects of medication, this could explain the different adaptive responses to IH shown by older adults with or without diseases. In addition to this, previous studies concluded that low doses of hypoxia (%FiO_2_ 13–15%) might not be a sufficient stimulus to induce adaptive mechanisms [25], and it is important to note that most of the studies included in this review carried out interventions within this range.

### Effects of IH on Body Composition and Metabolic Parameters

Only two studies [32, 47] out of eleven found an added effect of IH in parameters related to body composition, specifically a decrease in body fat mass [32, 47] and an increase in fat-free mass [47]. An oxygen-deprived environment leads to a decrease in body composition with larger changes occurring with higher hypoxic stress [52]. Previous studies have concluded that IH could have beneficial effects on weight loss as a consequence of an increase in the basal metabolic rate [14] and in appetite reduction as a consequence of an increase in leptin levels (satiety hormone) and a decrease in ghrelin levels (hunger-stimulating hormone) [53]. Likewise, the carotid body chemo-receptors under hypoxic exposure stimulate ventilation and lead to sympathetic activation increasing metabolic demands [54]. However, in most of the studies included in this systematic review no changes were found. The magnitude of changes in body composition depend on various factors, such as the type of intervention [55], the severity of hypoxic dose (*i.e.*, %FiO_2_ and duration) [52], the level of physical activity and the nutritional intake of the participants [56], in addition to the individual adaptive response [52]. These issues have not been carefully reported or controlled in any of the above-mentioned studies, suggesting their possible influence.

Regarding metabolic parameters, none of the five studies that investigated these variables revealed significant differences between either passive [32, 36] or active [37, 40, 41, 46] IH and similar intervention in normoxia. Given the fact that the participants included in those experiments had healthy normative values for metabolic parameters, it is plausible that IH did not affect them. Illustratively, when pre-diabetics and healthy older adults followed a 3-week passive IH intervention, only the pre-diabetic individuals reported significant reductions in fasting glucose [51]. This may indicate that molecular regulation of glucose and lipid metabolism, as well as mitochondrial function and the physiological mechanism underlying the beneficial effects of IH, would be different in older adults with or without diseases. Additionally, our results also suggest that active IH does not lead to major additional effects on metabolic parameters. Presumably, in a group of untrained older individuals, adaptations to the delivery, transport, and intramyocellular metabolism of glucose and insulin signaling pathway may be sufficiently large with exercise training in normoxia [46].

### Effects of IH on Blood Pressure and Hematological Parameters

Blood pressure has been one of the most studied health-related outcomes among the studies included in this review. While IH has been demonstrated to provide beneficial effects on the blood pressure of hypertensive [57] and obese [58] individuals, mainly due to vasodilation of the arteries and a decrease in arterial stiffness concomitant with an increase in nitric oxide [59], none of the studies included in this review observed significant changes of systolic blood pressure in healthy older adults [29, 32, 36, 37, 41, 45]. Only one study from this review [36] showed a significant decrease of diastolic blood pressure in overweight individuals after an 8-week passive hypoxia intervention (three 1-h sessions per week) compared to normoxia. The severity of the hypoxic dose used in this study (SpO_2_ = 75%) could have played an important role in lowering diastolic blood pressure. In this vein, hypoxic dose was shown to be a determining factor in achieving beneficial effects with IH, when %FiO_2_ ranged between 10 and 12% [25, 27]. Additionally, the fact that some older adults might be resistant to exercise-induced blood pressure reduction [60] can also influence the results obtained after an IH intervention.

Regarding hematological parameters, contradictory results were presented in the only two studies available: the first of these found increases in red-blood cells, hemoglobin and hematocrit after 12 sessions (3 sessions per week) of 120 min of IH [39], while the most recent one found no added effect of IH (*i.e.*, 5 weeks, 4 sessions per week, 60 min of resistance training) on hemoglobin, erythropoietin and blood volume [29]. It is noteworthy that, in the absence of total hemoglobin mass (tHb_mass_) measurement, the changes in hematological parameters must be interpreted with caution, as the increases reported could be the consequence of diuresis and plasma volume reduction. Previous studies have concluded that changes in hematological parameters will depend on both the total duration of the intervention and the session time [61]. In this vein, Gore et al. [62] stated that tHb_mass_ increases by 1.1% per 100 h of hypoxic exposure. On the other hand, not all participants could be considered ‘good’ responders, due to the large individual variability in the erythropoietic response to hypoxia [63].

### Effects of IH on Bone Parameters

Three studies focused on bone parameters [32, 35, 42]. In the analysis of bone health, it is recommended to use both static biomarkers [bone mineral content (BMC) and bone mineral density (BMD)], which provide information on long-term adaptations, and dynamic bone remodeling biomarkers [beta C-terminal telopeptide of collagen (b-CTX) and N-terminal propeptide of type I procollagen (PINP)] that are related to the rate of bone turnover and short-term adaptations [64]. In connection with BMD, only one study [35] observed significant improvements in whole body BMD, but not in femoral or trochanter BMD after 18 weeks of passive IH (%FiO_2_ 16.1%, two weekly 16-min sessions) in comparison with similar intervention in normoxia. However, these results must be interpreted with caution since an unclear risk of bias of blinding of participants and incomplete outcome data were detected in this study, in addition to the fact that the sample size was very small (n = 5 in the hypoxic group). In the other two studies, no change in whole body BMD was observed after 18 weeks of active IH (%FiO_2_ 16.1%) combined with WBV [42] or after 24 weeks of passive IH (%FiO_2_ 16.1%, three weekly 45-min sessions) [32]. In fact, previous scientific literature has stated that alterations in bone mass in the elderly require long-term physical exercise programs maintained over time [65], in addition to appropriate nutritional intake containing a good supply of proteins, minerals and vitamin D [66]. However, Timon et al. [32] observed both a significant increase of PINP (bone formation biomarker) and a significant decrease of b-CTX (bone resorption biomarker) after 24 weeks of passive IH intervention compared to normoxic intervention. The upregulation of HIF-1α that occurs under IH could activate different genes involved in bone remodeling, such as vascular endothelial growth factor (VEGF), erythropoietin and osteoprotegerin [67]. Likewise, IH could modulate the mesenchymal stem cells differentiation leading to a possible inhibition of bone resorption by increasing the osteoprotegerin/receptor activator [35].

### Effects of IH on Inflammatory Biomarkers

The influence of IH on inflammatory parameters has been scarcely investigated. To date, a 24-week intervention of passive IH exposure (%FiO_2_ 16.1%) produced a decrease in C-reactive protein levels compared to a control group, with no changes in IL-8 and IL-10 levels [32]. However, in another study [36] included in this review, no change in C-reactive protein levels was observed after an 8-week intervention of passive hypoxia. Previous studies have suggested that IH protocols could exert an anti-inflammatory and tissue-protective effects [68, 69], notably via the suppression of pro-inflammatory mediators such as TNF-α and IL-4 [70] and contribution to the production of anti-inflammatory interleukins by B cells [71]. However, when using active IH, such as resistance training with elastic bands during a 24-week intervention [34], no additive effect on inflammatory biomarkers was observed. It has been stated that the myokines produced during normoxic exercise have a long-term positive anti-inflammatory effect in older people [72, 73]. Supposedly, the anti-inflammatory role of exercise could mask the beneficial effect of the IH without any observed added effect on inflammatory parameters. Nonetheless, more evidence is needed to confirm the potential anti-inflammatory effect that passive or active IH could have on healthy older adults.

### Effects of IH on Functional Fitness, Pulmonary Capacity, and Quality of Life

Eight studies evaluated physical fitness [29, 33, 38, 39, 43–45, 47], but only one of them [47] observed improvements in pulmonary capacity (FVC, FEV_1_ and MVV) and functional fitness (*i.e.*, chair stand, pegboard, tandem test and one leg standing) in healthy older individuals when IH intervention was compared with normoxia. Park et al. [47] stated that this improvement was due to the greater aerobic and anaerobic exercise capacity and muscular function required by IH training *vs*. normoxic training. However, and despite the fact that some other studies have shown that IH could be beneficial for improving health-related fitness in healthy older adults [28] (albeit in the absence of comparison with a normoxic training group in this study) or those with cardiopathologies [50], the majority of the active IH experiments that used either resistance exercises [29, 33, 38], aerobic exercise [39, 45] or WBV [43, 44], indicated that the potential improvement obtained was not significantly greater than when using normoxic training. These contradictory findings may be explained by some methodological differences (*i.e.*, design of the training program, hypoxic dose). Unlike the other interventions, Park et al. [47] used a multimodal training program that combined aerobic work (treadmill and bicycle) with full-body muscular resistance training within the same session. This type of intervention has been shown to be very useful in older populations, both in normoxic [4, 74] and in hypoxic-hyperoxic conditions [75], to maintain or improve their physical and functional capacity. Additionally, it should be noted that the intensity of exercise used in this study was moderate-to-high, both in aerobic exercises (60–70% of maximal heart rate) and in resistance exercises (rating of perceived exertion-RPE of 6–7 out of 10). As suggested in previous studies [29, 76], low-to-moderate intensity or load may not be optimal to elicit molecular and structural adaptation. Nonetheless, similar absolute exercise intensity (*e.g.,* exercising at 100W) implies a higher relative workload when training in hypoxia than in normoxia, leading to greater physiological and perceptual responses [77, 78]. This variable should also be taken into account when analyzing the heterogeneous results of the studies, since some research has reported that if training in normoxia is carried out at the same workload as in hypoxia, no significant effects are observed [79].

Finally, It is also noteworthy that the hypoxic dose used by Park et al. [47] represents the highest training volume of all the included studies (36 sessions, 3 sessions per week, 90–120 min, %FiO_2_ 14.5%), which would indicate that long-term intervention of moderate IH and higher number of sessions could lead to greater benefits for functional fitness in the elderly.

Concerning quality of life and cognitive function, the results obtained are limited. Only two studies conducted by Schega et al. [38, 39] analyzed parameters related to these health-related outcomes. It was observed that 6 weeks (3 sessions per week) of 60-min passive IH followed by 30-min active IH (full-body resistance exercises at SpO_2_ of 80%) caused a positive added effect on attention and sleep quality compared with normoxic intervention, but not on the speed of cognitive performance or the mental component related to quality of life [38]. Likewise, 4 weeks (3 sessions/week) of passive IH (90 min) followed by 30 min of active IH (aerobic exercise on bicycle at SpO_2_ = 80%) also did not lead to any added benefit on cognitive function [39]. A recent systematic review has claimed that moderate hypoxia (depending on the type, severity, exposure duration and frequency) could have potential therapeutic applications for neurodegenerative diseases, such as mild cognitive impairments or dementia [6]. However, Schega et al. [38] indicate that the training effects of physical activity seem to outweigh the effect of IH which explains the ineffectiveness of IH on quality of life and cognitive performance in healthy older adults. More research is warranted to confirm this speculative hypothesis.

### Limitations

This systematic review is not without limitations. Firstly, only full articles written in English were reviewed, and two articles were not included in the review because they were written in Russian. Secondly, although most of the included studies (9 of 17) had a *low risk* of bias, several studies had a *high risk* or *unclear risk* of bias in some of the domains analyzed according to the Cochrane Collaboration recommendations. Thirdly, the included studies did not differentiate men and women, and a possible sex-related effect may have influenced the results. Fourthly, no studies concerning hypobaric hypoxia were included in this review, which may lead to different physiological responses than normobaric hypoxia. Finally, a meta-analysis could not be performed because the included studies presented a high diversity of interventions and great heterogeneity in health-related outcomes.

## Conclusions

IH has been suggested as potentially useful in older pathological individuals. However, this systematic review indicates that passive and active moderate IH had a limited effect on health-related outcomes in healthy older adults, compared to similar intervention in normoxia. No clear benefit has been evidenced on cardio-metabolic, inflammatory, hematological, and cognitive parameters, or on BMD, BMC, and lean mass, with the magnitude of changes dependent on the type of intervention and the hypoxic dose’s severity among others. Only in specific settings (*i.e.,* intermediate- or long-term interventions with high intensity/volume training sessions repeated at least 3 days per week under moderate-to-high hypoxic stress), may IH elicit optimal HIF-1α upregulation and consequent molecular and structural adaptations. In such conditions, some putative effect has been observed on functional fitness and fat mass loss. Nevertheless, more research is needed to determine the dose–response of passive/active IH in healthy older adults, especially to elucidate which factors are more decisive in explaining the individual variability of the response to IH.

## Data Availability

Not applicable.

## Abbreviations

1RM: One-repetition maximum
b-CTX: Beta C-terminal telopeptide of collagen
BDNF: Brain-derived neurotrophic factor
BMC: Bone mineral content
BMD: Bone mineral density
BP: Blood pressure
CHO: Cholesterol
CRP: C-reactive protein
DXA: Dual-energy X-ray absorptiometry
FEV1: Forced expiratory volume in 1 s
FiO_2_: Fraction of inspired oxygen
FVC: Forced vital capacity
GLUT4: Glucose transporter 4
Hb: Hemoglobin
Hct: Hematocrit
HDL: High-density lipoprotein
HIF: Hypoxia-inducible factor
HOMA-IR: Homeostatic model assessment-insulin resistance
HRmax: Maximum heart rate
HRV: Heart rate variability
IH: Intermittent hypoxia
MVV: Maximum voluntary ventilation
OSAS: Obstructive sleep apnea syndrome
PINP: N-terminal propeptide of type I procollagen
RCT: Randomized controlled trial
RPE: Rating of perceived exertion
SFT: Senior fitness test
SpO_2_: Peripheral oxygen saturation
TUGT: Timed up and go test
VCAM1: Vascular cell adhesion molecule 1
VEGF: Vascular endothelial growth factor
VO_2_max: Maximal oxygen uptake
WBV: Whole body vibration

## References

[1] Srivastava S (2017). The mitochondrial basis of aging and age-related disorders. Genes..

[2] Sun N, Youle RJ, Finkel T (2016). The mitochondrial basis of aging. Mol Cell.

[3] Shad BJ, Wallis G, van Loon LJ, Thompson JL (2016). Exercise prescription for the older population: the interactions between physical activity, sedentary time, and adequate nutrition in maintaining musculoskeletal health. Maturitas.

[4] Cordes T, Bischoff LL, Schoene D, Schott N, Voelcker-Rehage C, Meixner C (2019). A multicomponent exercise intervention to improve physical functioning, cognition and psychosocial well-being in elderly nursing home residents: a study protocol of a randomized controlled trial in the PROCARE (prevention and occupational health in long-term care) project. BMC Geriatr..

[5] Mateika JH, El-Chami M, Shaheen D, Ivers B (2015). Intermittent hypoxia: a low-risk research tool with therapeutic value in humans. J Appl Physiol..

[6] Burtscher J, Mallet RT, Burtscher M, Millet GP (2021). Hypoxia and brain aging: Neurodegeneration or neuroprotection?. Ageing Res Rev.

[7] Millet GP, Debevec T, Brocherie F, Malatesta D, Girard O (2016). Therapeutic use of exercising in hypoxia: promises and limitations. Front Physiol.

[8] Verges S, Chacaroun S, Godin-Ribuot D, Baillieul S (2015). Hypoxic conditioning as a new therapeutic modality. Front Pediatr.

[9] Brocherie F, Millet GP (2020). Hypoxic exercise as an effective nonpharmacological therapeutic intervention. Exp Mol Med..

[10] Lee JW, Ko J, Ju C, Eltzschig HK (2019). Hypoxia signaling in human diseases and therapeutic targets. Exp Mol Med..

[11] Kumar H, Choi DK (2015). hypoxia inducible factor pathway and physiological adaptation: a cell survival pathway?. Mediators Inflamm.

[12] Chen PS, Chiu WT, Hsu PL, Lin SC, Peng IC, Wang CY (2020). Pathophysiological implications of hypoxia in human diseases. J Biomed Sci.

[13] Morin R, Goulet N, Mauger JF, Imbeault P (2021). Physiological responses to hypoxia on triglyceride levels. Front Physiol.

[14] Kayser B, Verges S (2021). Hypoxia, energy balance, and obesity: an update. Obes Rev.

[15] Wang H, Shi X, Schenck H, Hall JR, Ross SE, Kline GP (2020). Intermittent hypoxia training for treating mild cognitive impairment: a pilot study. Am J Alzheimers Dis Other Demen..

[16] Gangwar A, Paul S, Ahmad Y, Bhargava K (2020). Intermittent hypoxia modulates redox homeostasis, lipid metabolism associated inflammatory processes and redox post-translational modifications: benefits at high altitude. Sci Rep..

[17] Stavrou VT, Astara K, Tourlakopoulos KN, Papayianni E, Boutlas S, Vavougios GD (2021). Obstructive sleep apnea syndrome: the effect of acute and chronic responses of exercise. Front Med.

[18] Franklin KA, Lindberg E (2015). Obstructive sleep apnea is a common disorder in the population-a review on the epidemiology of sleep apnea. J Thorac Dis.

[19] Gottlieb DJ (2021). Sleep apnea and cardiovascular disease. Curr Diab Rep..

[20] Guscoth LB, Appleton SL, Martin SA, Adams RJ, Melaku YA, Wittert GA (2021). The association of obstructive sleep apnea and nocturnal hypoxemia with lipid profiles in a population-based study of community-dwelling australian men. Nat Sci Sleep.

[21] Prabhakar NR, Peng YJ, Nanduri J (2020). Hypoxia-inducible factors and obstructive sleep apnea. J Clin Invest.

[22] Zhuang Y, Zhao Z, Cheng M, Li M, Si J, Lin K (2022). HIF-1α regulates osteogenesis of periosteum-derived stem cells under hypoxia conditions. Front Cell Dev Biol.

[23] Musutova M, Weiszenstein M, Koc M, Polak J (2020). Intermittent Hypoxia Stimulates Lipolysis, But Inhibits Differentiation and de novo lipogenesis in 3T3-L1 cells. Metab Syndr Relat Disord..

[24] Avezov K, Aizenbud D, Lavie L (2018). Intermittent hypoxia induced formation of "endothelial cell-colony forming units (EC-CFUs)" is affected by ros and oxidative stress. Front Neurol.

[25] Serebrovska TV, Serebrovska ZO, Egorov E (2016). Fitness and therapeutic potential of intermittent hypoxia training: a matter of dose. Fiziol Zh..

[26] Xi L, Serebrovskaya TV (2012). Intermittent hypoxia and human diseases.

[27] Coppel J, Hennis P, Gilbert-Kawai E, Grocott MP (2015). The physiological effects of hypobaric hypoxia versus normobaric hypoxia: a systematic review of crossover trials. Extrem Physiol Med.

[28] Shatilo VB, Korkushko OV, Ischuk VA, Downey HF, Serebrovskaya TV (2008). Effects of intermittent hypoxia training on exercise performance, hemodynamics, and ventilation in healthy senior men. High Alt Med Biol.

[29] Törpel A, Peter B, Schega L (2020). Effect of resistance training under normobaric hypoxia on physical performance, hematological parameters, and body composition in young and older people. Front Physiol.

[30] Page MJ, McKenzie JE, Bossuyt PM, Boutron I, Hoffmann TC, Mulrow CD (2021). The PRISMA 2020 statement: an updated guideline for reporting systematic reviews. BMJ.

[31] Higgins J, Thomas J, Chandler J, Cumpston M, Li T, Page M (2019). Cochrane handbook for systematic reviews of interventions.

[32] Timon R, González-Custodio A, Vasquez-Bonilla A, Olcina G, Leal A (2022). Intermittent hypoxia as a therapeutic tool to improve health parameters in older adults. Int J Environ Res Public Health..

[33] Timon R, Camacho-Cardeñosa M, González-Custodio A, Olcina G, Gusi N, Camacho-Cardeñosa A (2021). Effect of hypoxic conditioning on functional fitness, balance and fear of falling in healthy older adults: a randomized controlled trial. Eur Rev Aging Phys Act.

[34] Timon R, Martínez-Guardado I, Camacho-Cardeñosa A, Villa-Andrada JM, Olcina G, Camacho-Cardeñosa M (2021). Effect of intermittent hypoxic conditioning on inflammatory biomarkers in older adults. Exp Gerontol.

[35] Camacho-Cardenosa M, Quesada-Gómez JM, Camacho-Cardenosa A, Leal A, Dorado G, Torrecillas-Baena B (2020). Effects of normobaric cyclic hypoxia exposure on mesenchymal stem-cell differentiation-pilot study on bone parameters in elderly. World J Stem Cells.

[36] Chacaroun S, Borowik A, Doutreleau S, Belaidi E, Wuyam B, Tamisier R (2020). Cardiovascular and metabolic responses to passive hypoxic conditioning in overweight and mildly obese individuals. Am J Physiol Regul Integr Comp Physiol.

[37] Chacaroun S, Borowik A, Gonzalez VEY, Doutreleau S, Wuyam B, Belaidi E (2020). Hypoxic exercise training to improve exercise capacity in obese individuals. Med Sci Sports Exerc.

[38] Schega L, Peter B, Törpel A, Mutschler H, Isermann B, Hamacher D (2013). Effects of intermittent hypoxia on cognitive performance and quality of life in elderly adults: a pilot study. Gerontology.

[39] Schega L, Peter B, Brigadski T, Leßmann V, Isermann B, Hamacher D (2016). Effect of intermittent normobaric hypoxia on aerobic capacity and cognitive function in older people. J Sci Med Sport.

[40] Allsopp GL, Addinsall AB, Hoffmann SM, Russell AP, Wright CR (2022). Hormonal and metabolic responses of older adults to resistance training in normobaric hypoxia. Eur J Appl Physiol.

[41] Allsopp GL, Hoffmann SM, Feros SA, Pasco JA, Russell AP, Wright CR (2022). The effect of normobaric hypoxia on resistance training adaptations in older adults. J Strength Cond Res.

[42] Camacho-Cardenosa M, Camacho-Cardenosa A, Burtscher M, Brazo-Sayavera J, Tomas-Carus P, Olcina G (2019). Effects of whole-body vibration training combined with cyclic hypoxia on bone mineral density in elderly people. Front Physiol..

[43] Camacho-Cardenosa M, Camacho-Cardenosa A, Brazo-Sayavera J, Olcina G, Tomas-Carus P, Timon R (2019). Evaluation of 18-week whole-body vibration training in normobaric hypoxia on lower extremity muscle strength in an elderly population. High Alt Med Biol..

[44] Camacho-Cardenosa M, Camacho-Cardenosa A, Tomas-Carus P, Olcina G, Timon R, Brazo-Sayavera J (2020). Effects of whole-body vibration under hypoxic exposure on muscle mass and functional mobility in older adults. Aging Clin Exp Res..

[45] Hein M, Chobanyan-Jürgens K, Tegtbur U, Engeli S, Jordan J, Haufe S (2021). Effect of normobaric hypoxic exercise on blood pressure in old individuals. Eur J Appl Physiol.

[46] Chobanyan-Jürgens K, Scheibe RJ, Potthast AB, Hein M, Smith A, Freund R (2019). Influences of hypoxia exercise on whole-body insulin sensitivity and oxidative metabolism in older individuals. J Clin Endocrinol Metab..

[47] Park HY, Jung WS, Kim J, Lim K (2019). Twelve weeks of exercise modality in hypoxia enhances health-related function in obese older Korean men: a randomized controlled trial. Geriatr Gerontol Int.

[48] Glazachev OS, Kryzhanovskaya SY, Zapara MA, Dudnik EN, Samartseva VG, Susta D (2021). Safety and efficacy of intermittent hypoxia conditioning as a new rehabilitation/ secondary prevention strategy for patients with cardiovascular diseases: a systematic review and meta-analysis. Curr Cardiol Rev.

[49] Behrendt T, Bielitzki R, Behrens M, Herold F, Schega L (2022). Effects of intermittent hypoxia-hyperoxia on performance- and health-related outcomes in humans: a systematic review. Sports Med Open.

[50] Burtscher M, Pachinger O, Ehrenbourg I, Mitterbauer G, Faulhaber M, Puhringer R (2004). Intermittent hypoxia increases exercise tolerance in elderly men with and without coronary artery disease. Int J Cardiol.

[51] Serebrovska TV, Portnychenko AG, Drevytska TI, Portnichenko VI, Xi L, Egorov E (2017). Intermittent hypoxia training in prediabetes patients: beneficial effects on glucose homeostasis, hypoxia tolerance and gene expression. Exp Biol Med.

[52] Dünnwald T, Gatterer H, Faulhaber M, Arvandi M, Schobersberger W (2019). Body composition and body weight changes at different altitude levels: a systematic review and meta-analysis. Front Physiol.

[53] Shukla V, Singh SN, Vats P, Singh VK, Singh SB, Banerjee PK (2005). Ghrelin and leptin levels of sojourners and acclimatized lowlanders at high altitude. Nutr Neurosci.

[54] Kara T, Narkiewicz K, Somers VK (2003). Chemoreflexes–physiology and clinical implications. Acta Physiol Scand.

[55] Davis ME, Blake C, Perrotta C, Cunningham C, O’Donoghue G (2022). Impact of training modes on fitness and body composition in women with obesity: a systematic review and meta-analysis. Obesity..

[56] Kietzmann T, Mäkelä VH (2021). The hypoxia response and nutritional peptides. Peptides.

[57] Muangritdech N, Hamlin MJ, Sawanyawisuth K, Prajumwongs P, Saengjan W, Wonnabussapawich P (2020). Hypoxic training improves blood pressure, nitric oxide and hypoxia-inducible factor-1 alpha in hypertensive patients. Eur J Appl Physiol.

[58] González-Muniesa P, Lopez-Pascual A, de Andrés J, Lasa A, Portillo MP, Arós F (2015). Impact of intermittent hypoxia and exercise on blood pressure and metabolic features from obese subjects suffering sleep apnea-hypopnea syndrome. J Physiol Biochem.

[59] Vedam H, Phillips CL, Wang D, Barnes DJ, Hedner JA, Unger G (2009). Short-term hypoxia reduces arterial stiffness in healthy men. Eur J Appl Physiol.

[60] Stewart KJ, Bacher AC, Turner KL, Fleg JL, Hees PS, Shapiro EP (2005). Effect of exercise on blood pressure in older persons: a randomized controlled trial. Arch Intern Med.

[61] Lobigs LM, Sharpe K, Garvican-Lewis LA, Gore CJ, Peeling P, Dawson B (2018). The athlete's hematological response to hypoxia: a meta-analysis on the influence of altitude exposure on key biomarkers of erythropoiesis. Am J Hematol..

[62] Gore CJ, Sharpe K, Garvican-Lewis LA, Saunders PU, Humberstone CE, Robertson EY (2013). Altitude training and haemoglobin mass from the optimised carbon monoxide rebreathing method determined by a meta-analysis. Br J Sports Med.

[63] Jedlickova K, Stockton DW, Chen H, Stray-Gundersen J, Witkowski S, Ri-Li G (2003). Search for genetic determinants of individual variability of the erythropoietin response to high altitude. Blood Cells Mol Dis..

[64] Brown J, Albert C, Nassar B, Adachi J, Cole D, Davison K (2009). Bone turnover markers in the management of postmenopausal osteoporosis. Clin Biochem..

[65] Gómez-Cabello A, Ara I, González-Agüero A, Casajús JA, Vicente-Rodríguez G (2012). Effects of training on bone mass in older adults: a systematic review. Sports Med.

[66] Rizzoli R, Biver E, Brennan-Speranza TC (2021). Nutritional intake and bone health. Lancet Diabetes Endocrinol..

[67] Wu C, Rankin E, Castellini L, Fernandez-Alcudia J, LaGory E, Andersen R (2015). Oxygen-sensing PHDs regulate bone homeostasis through the modulation of osteoprotegerin. Genes Dev.

[68] Serebrovska ZO, Xi L, Tumanovska LV, Shysh AM, Goncharov SV, Khetsuriani M (2022). Response of circulating inflammatory markers to intermittent hypoxia-hyperoxia training in healthy elderly people and patients with mild cognitive impairment. Life.

[69] Kiers D, Wielockx B, Peters E, van Eijk LT, Gerretsen J, John A (2018). Short-term hypoxia dampens inflammation in vivo via enhanced adenosine release and adenosine 2B receptor stimulation. EBioMedicine.

[70] Serebrovskaya TV, Nikolsky IS, Nikolska VV, Mallet RT, Ishchuk VA (2011). Intermittent hypoxia mobilizes hematopoietic progenitors and augments cellular and humoral elements of innate immunity in adult men. High Alt Med Biol.

[71] Meng X, Grötsch B, Luo Y, Knaup KX, Wiesener MS, Chen XX (2018). Hypoxia-inducible factor-1α is a critical transcription factor for IL-10-producing B cells in autoimmune disease. Nat Commun..

[72] Bautmans I, Salimans L, Njemini R, Beyer I, Lieten S, Liberman K (2021). The effects of exercise interventions on the inflammatory profile of older adults: a systematic review of the recent literature. Exp Gerontol.

[73] Chupel MU, Direito F, Furtado GE, Minuzzi LG, Pedrosa FM, Colado JC (2017). Strength training decreases inflammation and increases cognition and physical fitness in older women with cognitive impairment. Front Physiol.

[74] Lopez P, Pinto RS, Radaelli R, Rech A, Grazioli R, Izquierdo M (2018). Benefits of resistance training in physically frail elderly: a systematic review. Aging Clin Exp Res.

[75] Bayer U, Glazachev OS, Likar R, Burtscher M, Kofler W, Pinter G (2017). Adaptation to intermittent hypoxia-hyperoxia improves cognitive performance and exercise tolerance in elderly. Adv Gerontol.

[76] Vogt M, Puntschart A, Geiser J, Zuleger C, Billeter R, Hoppeler H (2001). Molecular adaptations in human skeletal muscle to endurance training under simulated hypoxic conditions. J Appl Physiol..

[77] Li Y, Li J, Atakan MM, Wang Z, Hu Y, Nazif M (2022). Methods to match high-intensity interval exercise intensity in hypoxia and normoxia—a pilot study. J Exerc Sci Fit.

[78] Pramsohler S, Burtscher M, Faulhaber M, Gatterer H, Rausch L, Eliasson A (2017). Endurance training in normobaric hypoxia imposes less physical stress for geriatric rehabilitation. Front Physiol.

[79] Desplanches D, Hoppeler H, Linossier MT, Denis C, Claassen H, Dormois D (1993). Effects of training in normoxia and normobaric hypoxia on human muscle ultrastructure. Pflugers Arch.

